# Prevalence of hypertension among adult outpatient clients in hospitals and its associated factors In Addis Ababa, Ethiopia: a hospital based cross-sectional study

**DOI:** 10.1186/s13104-019-4127-1

**Published:** 2019-02-14

**Authors:** Selamawit Abebe, Walelegn Worku Yallew

**Affiliations:** 1 Addis Continental Institute of Public Health, Addis Ababa, Ethiopia; 2 Department of Environmental and Occupational Health, College of Public Health, Institute of Public Health, University of Gondar, Po. Box 196, Gondar, Ethiopia

**Keywords:** Hypertension, Prevalence, Physical inactivity, Ethiopia

## Abstract

**Objective:**

The objective of the research is to measure prevalence of hypertension and associated factors adult outpatient clients in Addis Ababa, Ethiopia.

**Results:**

A total of 487 participants were included in the study. The prevalence of hypertension was (34.7%), 95% CI (30.6–38.8), of them 53.8 were Male and 46.2% were female. Among 169 participants identified as having high blood pressure, 131 (66.5%) were aware of their blood pressure, from those 96 (48.7%) of them were receiving antihypertensive medication. Mean elevated systolic blood pressure was 124.7 ± 20 (SD) and diastolic blood pressure was 81.5 ± 9.8 (SD). The multivariable regression analysis showed that having ever been told hypertension (AOR = 15.47, 7.74–30.89); using animal product butter (AOR = 2.66, 1.25–5.67); physical inactivity (AOR = 2.83, 1.34–6.01) and BMI 25.0 to 29.9 and greater than 30 (AOR = 5.02, 1.58–15.94 and AOR = 3.98, 1.04–16.01, respectively) were statistically significant predictors of hypertension. The prevalence of hypertension was considerably high. The health system needs to develop strategies to increase the reach of relevant screening and diagnostic services.

**Electronic supplementary material:**

The online version of this article (10.1186/s13104-019-4127-1) contains supplementary material, which is available to authorized users.

## Introduction

Hypertension accounts for approximately 50% of coronary heart disease and 67% for the cerebra vascular disease burden worldwide [1]. It is one of public health challenges, which affects approximately one billion people, around half of all stroke and heart disease related deaths [2]. In the first half of the twentieth century, hypertension was almost non-existent in African. However, evidence shows that more than 40% of African adults have hypertension currently [3].

Hypertension is not only an important public health problem; rather, it will also have a big economic impact [4]. Many factors contribute to the high prevalence rates of hypertension including eating food containing too much salt and fat; not eating enough fruits and vegetables [5].

World Health Organization (WHO) urges countries to develop measures and strengthen health care services to reduce the level of exposure risk factors for non communicable diseases (NCDs) [5]. In well-developed primary health-care system settings, adding an organized screening programme to usual practice may not be required. Accessing systematic screening of the population healthcare settings reduces the incidence of ischemic heart disease [6].

In sub-Saharan Africa (SSA), hypertension and other cardiovascular diseases were not given due attention [7]. An increasing burden of hypertension in this region will thus result in grave consequences, as only very few people get treatment [8]. The epidemiology of hypertension in Ethiopia is not well studied. Therefore, evidences are required in prevalence and associated factors to raise the knowledge of the population and policymakers for appropriate design and implementation on prevention, treatment and control of hypertension in the country. There for this study was conducted to measure prevalence of hypertension and associated factors adult outpatient clients in Addis Ababa.

## Main text

### Methods

The study was conducted at Yekatit 12 hospital in Addis Ababa, Ethiopia. A hospital based cross sectional study conducted from Nov to Dec 2015 in Addis Ababa, Ethiopia.

Simple random sampling procedure was conducted in outpatient departments. A total of 487 outpatient department clients participated in this research whose age ≥ 18 years. Patients with serious illnesses, acute life-threatening conditions, and severe injury were excluded from the study.

Data was collected using a structured interview questionnaire and physical measurements which was adapted from “WHO STEPS’’ tool that contain Socio-demographic, behavioural, and other variables [9]. A digital measuring instrument (Seca 700 weight scale, Germany) was used to measure the weight of adult individuals who were included in the study. Weight measuring scales was checked and adjusted at zero level before each measurement. Height was measured with stadiometers following the standard steps [9–12].

Blood pressure (BP) was measured twice in a sitting position (using a standard sphygmomanometer BP cuff with an appropriate size to cover two-thirds of the upper arm) after the participant rested for at least 5 min, the second measurement was taken 5–10 min after the first measurement. Participants were inquired whether they had consumed any hot beverage such as tea or coffee, smoked cigarette or undertaken any physical activity 30 min before measurement. Hypertension defined as systolic blood pressure ≥ 140 mmHg and/or diastolic blood pressure ≥ 90 mmHg or treatment with antihypertensive agents [9–11].

To ensure the data quality, there was 2-day training for the data collectors and supervisors. Pre-test to see the effectiveness of the questionnaire and if there were additional needs, continuous and strict supervision was conducted on the spot.

Data was entered to Epidemiological Information (Epi Info) version 3.5 and analysed by IBM^®^ SPSS Statistics software version 20. Descriptive statistics such as frequency and cross tabulation were calculated for selected variables. Multivariable logistic regression model was used to identify factors associated with hypertension. Adjusted odds ratio (AOR) with 95% confidence interval (CI) and P-value ≤ 0.05 in the final model were used to determine significant predictors.

### Results

A total of 487 clients participated in the study with 100% response rate. The mean age of the participants was 46.71 with the standard derivation of 15.67. The majority, 380 (78%) were Orthodox and 294 (60.4%) were married. About 94 (13.5%) had no formal education (Table 1).

**Table 1:** Socio demographic characteristics of adult outpatient clients in Yekatit 12 Hospital, Addis Ababa, Ethiopia, 2016

Variable	Frequency	Percent (%)
Sex		
Male	237	48.7
Female	250	51.3
Total	487	100.0
Age group		
18–27	57	11.7
28–37	96	19.7
38–47	102	20.9
48–57	88	18.1
58+	144	29.6
Total	487	100.0
Religion		
Orthodox	380	78
Muslim	54	11.1
Protestant	41	8.4
Catholic	2	0.4
Others	10	2.1
Total	487	100.0
Ethnicity		
Amhara	245	50.3
Oromo	114	23.4
Tigre	36	7.4
Gurage	57	11.7
Others	35	7.2
Total	487	100.0
Marital status		
Single	112	23
Married	294	60.4
Separated	17	3.5
Divorced	15	3.1
Widowed	49	10.1
Total	487	100.0
Educational status		
No formal education	94	19.3
Primary school (1–8)	165	33.9
Secondary school (9–12)	142	29.2
College or university	83	17
Post graduate and above	3	0.6
Total	487	100.0

From 487 participants 146 (30%) had family history of hypertension. Of all 430 (88.3%) which were measured their blood level by the health professional they know their hypertension status. From these 96 (48.7%) take antihypertensive medicine currently. Among all 487 respondents 96 (19.7%) were take antihypertensive medicine currently. Only 1/4th of the participants, 121 (24.8%) had family history of diabetes. Ninety-two 18.9% had past history of tobacco smoking. The habit of eating fruits of the respondents was satisfactory, which 228 (46.8%) of them ate fruit 1–2 days per week, 166 (34.1%) less than 1 day per week. Only 155 (31.8%) of the respondents were participate daily on vigorous working activity.

The overall prevalence of hypertension was 169 (34.7%) with 95% CI 30.6–38.8. Among 169 participants identified as having high blood pressure, 131 (66.5%) were aware of their blood pressure and 96 (48.7%) of them were receiving antihypertensive medication, of which 16 (16.7%) had normal (controlled) blood pressure at the time of the study.

In the multivariable logistic regression analysis, having ever been told hypertension; using animal product butter; physical inactivity and BMI were statistically significant predictors of hypertension. The odds of developing hypertension among respondents who had ever been told hypertension was 15.47-times more likely compared to the counterparts who had not (AOR = 15.47, 95% CI 7.74–30.89).

Using animal product butter type was the other significant predictor of hypertension, so that respondents who ate animal product butter type were 2.66-times more likely to develop hypertension as compared to those who did not use any type of butter (AOR = 2.66, 95% CI 1.25–5.67). Also, respondents who did not physical exercise were 2.83-times more likely to have hypertension compared to those who did physical exercise (AOR = 2.83, 95% CI 1.34–6.01).

Moreover, the BMI of the respondents was statistically associated with developing hypertension, the odds of hypertension being more than four-, five- and three-times higher among respondents whose BMI ranged from 18.5 to 24.9, 25.0 to 29.9 and greater than 30.0 compared to whose BMI was less than 18.5, respectively (AOR = 4.08, 95% CI 1.33–12.51, AOR = 5.02, 95% CI 1.58–15.94 and AOR = 3.98, 95% CI 1.04–16.01), respectively (Table 2).

**Table 2:** Bivariate and Multivariate analysis for risk factors of hypertension among Out Patient Clients in Yekatit 12 Hospital, Addis Ababa, Ethiopia

Variable	Yes (%)	No (%)	COR (95% CI)	AOR (95% CI)
Age group				
18–27	5 (8.8%)	52 (91.2%)	1.00	1.00
28–37	20 (20.8%)	76 (79.2%)	2.73 (0.96–7.75)	1.19 (0.33–4.3)
38–47	28 (27.5%)	74 (72.5%)	3.93 (1.42–10.86)*	1.02 (0.29–3.53)
48–57	40 (45.5%)	48 (54.5%)	8.66 (3.15–23.77)**	1.68 (0.45–6.21)
58+	76 (52.8%)	68 (47.2%)	11.62 (4.38–30.79) **	2.44 (0.7–8.49)
Having ever been told hypertension				
Yes	131 (66.5%)	66 (33.5%)	13.16 (8.38–20.67)**	15.47 (7.74–30.8)*
No	38 (13.1%)	252 (86.9%)	1.00	1.00
Having ever been told diabetes				
Yes	63 (11.6%)	59 (48.4%)	2.6 (1.71–3.97)**	0.63 (0.26–1.52)*
No	106 (29%)	259 (71%)	1.00	1.00
Past tobacco smoking				
Yes	46 (50%)	46 (50%)	2.21 (1.39–3.5)**	1.8 (0.87–3.69)
No	123 (31.1%)	272 (68.9%)	1.00	1.00
Past alcohol drinking				
Yes	125 (41.1%)	179 (58.9%)	2.2 (1.46–3.32)**	0.83 (0.4–1.75)
No	44 (24%)	139 (76%)	1.00	1.00
Current alcohol drinking				
Yes	82 (43.2%)	108 (56.8%)	1.83 (1.253–2.68)**	1.69 (0.49–5.83)
No	87 (23.3%)	210 (70.7%)	1.00	1.00
Butter type				
Animal product	111 (40.4%)	164 (59.6%)	1.51 (1.89–2.5)*	2.66 (1.25–5.67)*
Vegetable product	32 (25%)	96 (75%)	0.74 (0.4–1.37)	1.04 (0.44–2.44)
Do not use	26 (31%)	58 (69%)	1.00	1.00
Participate on vigorous working activity				
Yes	42 (27.1%)	113 (72.9%)	1.00	1.00
No	127 (38.3%)	205 (61.7%)	1.66 (1.09–2.53)**	0.85 (0.46–1.59)
Doing physical exercise/sport				
Yes	20 (23.8%)	64 (76.2%)	1.00	1.00
No	149 (37%)	254 (63%)	1.87 (1.09–3.22)**	2.83 (1.34–6.01)*
Body mass index				
Underweight	7 (13.79%)	44 (86.3%)	1.00	1.00
Normal	79 (30.3%)	182 (69.7%)	2.72 (1.17–6.32)*	4.08 (1.33–12.51)*
Overweight	66 (47.8%)	72 (52.2%)	5.76 (2.42–13.68)**	5.02 (1.58–15.94)*
Obese	17 (45.9%)	20 (54.1%)	5.34 (1.91–14.91)**	3.98 (1.04–16.11)*

* p-value < 0.05, ** p-value < 0.005

### Discussion

The proportion of hypertension among outpatient’s clinic was 34.7% among outpatient visitors. More than half of hypertension cases was untreated and the control rate was very low. Unaware of hypertension, overweight, obesity, eating animal product like butter and physical inactivity were statistically significant.

The overall prevalence of hypertension was 34.7%, which is significantly higher than both a hospital based [13] and a community based cross sectional studies [14–18] done in the country. This discrepancy could be explained in three ways; this study is a hospital based that the participants were patients, whereas the previous studies were a community based. The second reason for the discrepancy might be the age difference in the study population (≥ 18 years of age with the mean age of 46.7 in this case were participated while other study included aged ≥ 15 years [13], 25–64 years [16] and > 31 years [15]. And the other reason for this study is considered only urban setting whereas the former studies included urban and rural settings [15, 17, 18]. This coincides well with findings in most studies conducted in SSA, where the prevalence rate of hypertension was found to be higher in urban dwellers than in rural dwellers [8].

This study revealed a considerably high prevalence of overweight and obesity among adult outpatient clients. Only one-third of the population worked on vigorous working activity and most of them were not physically active. Urbanization is associated with changes in dietary habits and with reduced physical activity that lead to obesity. Such changes of lifestyle and dietary habits contribute to overweight and obesity in urban areas which eventually result in the increased prevalence of hypertension [8]. Ethiopia Demographic and Health Survey (EDHS) indicate that urban women and men are more likely to be overweight or obese than rural women and men. One woman of every five residing in Addis Ababa are overweight [19]. We enrolled females in the age of 18 and above, while the EDHS included women 15–49 years of age in Addis. Thus, higher mean age of our study participants might have contributed to the higher overall prevalence of overweight.

This finding is similar with the prevalence in Southern Africa (34.6%) and Northern Africa (33.3%) [20]. Also, studies conducted in urban Namibia 35.9% [8] and higher than the study conducted in Nigerian Missionary Hospital 30.6% were similar [21]. This finding is lower than a study in Kazakhstan (70%) [22], the higher result may be due to older age populations (50–75 years age). Among subjects who were identified as having hypertension, 33.5% were unaware of their blood pressure, indicating that, one out of three cases of hypertension do not know that they have it. This supports the notion that hypertension is a silent killer. This finding was in line with that of study done on urban dwellers of Durame and Bahir Dar which were 37.8% and 38.6%, respectively [15, 18], but much differ than a study done in Addis Ababa (64.8%) [16]. From those 48.7% who were receiving antihypertensive medication only 16.7% had normal blood pressure at the time of the study. The finding is alarming in that the burden of uncontrolled hypertension is huge and requires urgent attention.

More than one-third of adults was overweight/obese (35.9%) in this study; and 47.4% of them were hypertensive which was consistent with findings reported in community-based studies in Sub-Saharan Africa countries. Moreover, BMI was statistically associated with hypertension, with the odds of hypertension being more than five- and four-times higher among respondents who were overweight and obese as compared to respondents whose BMI was less than 18.5. This finding is in accordance with studies conducted in our country and other countries in the world, where higher BMI was associated with hypertension [8, 16, 17, 23–25].

Respondents who didn’t do exercise/sports were more than 2.83-times more likely to have hypertension compared to those who did physical exercise. The prevalence of physical activity in this study is (17.2%) which is higher than the study conducted in Bahirdar (4.9%) [18] but much lower than study conducted in urban Kenya [24]. Using animal product butter type was associated with hypertension, so that respondents who ate animal product butter type were 2.66-times more likely to develop hypertension as compared to those who did not use any type of butter.

The prevalence of hypertension was found to be high among Outpatient clients. Awareness, treatment, and control rates in those with the disease were low this supports the notion that hypertension is a silent killer. Factors like physical inactivity, family history of hypertension, eating animal product butter type and being overweight and obesity were found to be associated with high BP whereas, sex, vegetable and fruit consuming not associated with high blood pressure. The findings in this study and other recent studies conducted in the country have clearly shown that hypertension is becoming a serious public health concern. Therefore, mass screening of hypertension and life style modifications and motivating the community for adequate BP control are recommended. It is also better to give special emphasis for health education regarding the daily live events like healthy dietary habit and regular exercise and preventing alcohol consumption and cigarette smoking. Policy makers need to focus on community level intervention through integration with the open-door health extension program. Furthermore, there must be national strategy for hypertension screening at least for adults visiting health institutions for various reasons.

## Limitation

This finding is not free from limitations; most of our patients don’t knew their exact birth date, as a result there was no reliable account of their age. Secondly, which was based on only self-reports of a previous diagnosis and history of personal history of diabetes, alcohol drinking, cigarette smoking. This study may lack generalizability for the community at large.

## Additional file

**Additional file 1.** Prevalence of hypertension data. The prevalence of hypertension and factors associated to it.

## Abbreviations

AOR: adjusted odds ratio
BMI: body mass index
BP: blood pressure
CI: confidence interval
EDHS: Ethiopia Demographic and Health Survey
Epi Info: Epidemiological Information
NCD: non-communicable diseases
SD: standard deviation
SSA: Sub-Saharan Africa
WHO: World Health Organization
